# The Evolutionary Volte-Face of Transposable Elements: From Harmful Jumping Genes to Major Drivers of Genetic Innovation

**DOI:** 10.3390/cells10112952

**Published:** 2021-10-29

**Authors:** Melody Nicolau, Nathalie Picault, Guillaume Moissiard

**Affiliations:** 1 LGDP-UMR5096, CNRS, 66860 Perpignan, France; melody.nicolau@univ-grenoble-alpes.fr (M.N.); nathalie.picault@univ-perp.fr (N.P.); 2 LGDP-UMR5096, Université de Perpignan Via Domitia, 66860 Perpignan, France

**Keywords:** transposable elements, silencing, epigenetics, exaptation

## Abstract

Transposable elements (TEs) are self-replicating DNA elements that constitute major fractions of eukaryote genomes. Their ability to transpose can modify the genome structure with potentially deleterious effects. To repress TE activity, host cells have developed numerous strategies, including epigenetic pathways, such as DNA methylation or histone modifications. Although TE neo-insertions are mostly deleterious or neutral, they can become advantageous for the host under specific circumstances. The phenomenon leading to the appropriation of TE-derived sequences by the host is known as TE exaptation or co-option. TE exaptation can be of different natures, through the production of coding or non-coding DNA sequences with ultimately an adaptive benefit for the host. In this review, we first give new insights into the silencing pathways controlling TE activity. We then discuss a model to explain how, under specific environmental conditions, TEs are unleashed, leading to a TE burst and neo-insertions, with potential benefits for the host. Finally, we review our current knowledge of coding and non-coding TE exaptation by providing several examples in various organisms and describing a method to identify TE co-option events.

## 1. Introduction

In eukaryotic cells, the chromatin fiber is precisely packaged and organized in subnuclear compartments that undergo dynamic spatial rearrangements depending on developmental transitions and environmental constraints [1,2,3,4]. Actively transcribed protein-coding genes compose the euchromatin, which is a relaxed form of chromatin, while spatiotemporally regulated genes that are transcriptionally repressed, such as developmental genes, form facultative heterochromatic foci [5]. Finally, the constitutive heterochromatin is highly condensed and enriched in DNA repeats, including silenced transposable elements (TEs) [6,7,8].

Upon their discovery in the 1940s, Barbara McClintock defined TEs as “controlling elements […] operating as an integrated system in the control of gene action” [9]. Subsequently, they received the names of “jumping” genes, as well as selfish, parasitic or junk DNA [10,11], most likely because TEs are highly repeated, self-replicating mobile elements, capable of invading the host genome by a mechanism called transposition. Indeed, TEs constitute a significant fraction of plant genomes, ranging from 15% in *Arabidopsis thaliana* up to 85% in maize [12,13,14]; in animals, they represent half of the human nuclear DNA content, and one third of the *Drosophila melanogaster* genome [15]. Altogether, this excessive junk DNA, including TEs and other DNA repeats, can greatly explain the C-value paradox that Freeling and colleagues proposed to solve by hypothesizing that bulk junk DNA would serve as “genome balance” [16].

TEs are classified in two major classes depending on their mode of transposition. Class I TEs (or retrotransposons) mobilize via an RNA intermediate in a “copy-and-paste” strategy, while class II TEs (or DNA transposons) mobilize via a DNA intermediate in a “cut-and-paste” strategy [17]. Each class is composed of several subclasses, superfamilies and families gathering TEs with similar features [17,18,19]. Among class I TEs, the two major subclasses are long terminal repeat (LTR) and non-LTR retrotransposons [13]. Among class II TEs, there are terminal inverted repeat (TIR) and non-TIR DNA transposons and rolling-circle (RC)/Helitrons [12,13,17]. Except for some families such as RC/Helitrons or Spy transposons, most TEs are flanked by target site duplication (TSD) repeats that are a few nucleotides generated by the DNA repair machinery after the TE neo-insertion. TEs subclasses often include autonomous and non-autonomous TEs, the latter using the functional transposition machinery of family-related autonomous TEs to invade the genome. For instance, among non-LTR retrotransposons, short interspersed nuclear elements (SINEs) use the long interspersed nuclear elements (LINEs) machinery to amplify themselves [20]. Similarly, it is now well established that Activator/Dissociation (Ac/Ds) elements, originally described by Barbara McClintock in 1950 [21], are autonomous and non-autonomous TIR DNA transposons, respectively.

Depending on the insertion site, neo-insertions of TEs can differently impact the host genome. They can first be neutral, for instance, if they happen in non-regulatory intergenic regions. They can also be deleterious, by disrupting the sequence of protein-coding genes, or provoking chromosomal rearrangement [18,22,23,24,25]. Because TEs are potentially harmful for the host, they need to be transcriptionally repressed. Several cellular processes, including epigenetic pathways like DNA methylation and histone modifications, serve the purpose of keeping TEs silenced [6,7,8]. Finally, in some cases, TE neo-insertions can turn into a beneficial outcome to the host, by providing an evolutionary advantage [24]. Through these beneficial insertions, TEs can create genetic diversity and innovation, contributing to genome evolution. Thus, despite their potentially deleterious nature, TEs can become beneficial to the host [25,26]. The host-TE interaction relies on a tight and complex relationship, from conflict (or arms race), cooperation (or equilibrium) to co-option (or TE exaptation) [27].

In this review, we will first describe the recent progress made in the field of TE silencing, with a focus on plants. In the second part, we will attempt to explain how harmful, parasitic TEs can turn into beneficial genetic elements that contribute to evolutionary innovation of the host genome. Finally, we will describe different approaches that exist to study host-TE interaction, and to bring to light genuine events of TE exaptation.

## 2. A Plethora of Sophisticated Epigenetic Pathways Dampen TEs

DNA methylation, histone modifications and histone variants and non-coding RNA (ncRNA), like small interfering RNA (siRNA), chromatin remodelers and other epigenetic factors, play important roles in many chromatin-related processes, including genome organization, DNA replication and repair, regulation of gene expression and TE silencing [7,28,29]. All these epigenetic pathways cooperate to efficiently silence TEs, forming a multilayered lock system that can be depicted as an epigenetic “mille-feuille” [30]. These epigenetic pathways can act synergistically or redundantly, depending on the targeted TEs. In addition, these multifaceted epigenetic pathways are tightly intertwined, and crosstalk is often observed, which makes overall TE silencing a combination of very sophisticated and complex molecular processes.

### 2.1. DNA Methylation, an Overview

**Figure 1:**
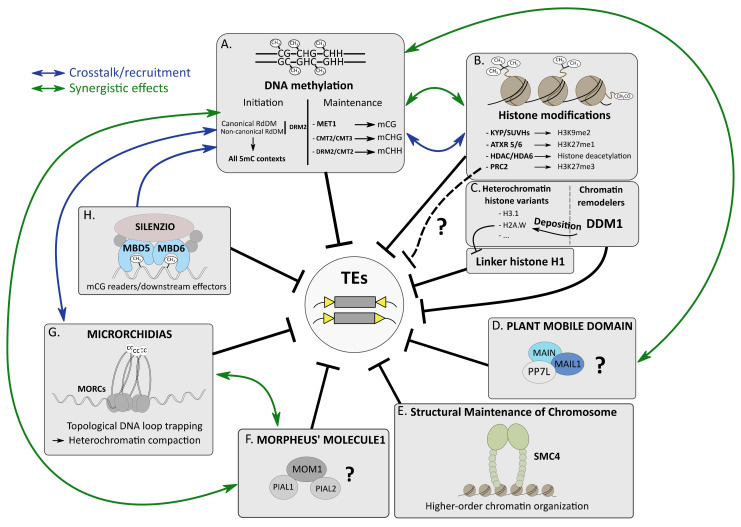
Several silencing pathways cooperate to silence TEs in *A. thaliana*. (**A**)**.** Initiation and maintenance of DNA methylation involve specific DNA methyltransferases to perform 5mC in three cytosine contexts. (**B**)**.** Histone modifications such as methylation of histone 3 at lysine 9 or lysine 27 involve specialized histone methyltransferases. Deacetylation of histone tails requires HDAC proteins, such as HDA6. (**C**)**.** DDM1 is involved in the deposition of the heterochromatic histone variant H2A.W, which interferes with linker histone H1 occupancy at constitutive heterochromatin. The connections between DDM1, H3K27me3 and 5mC remain unclear. (**D**)**.** PMD proteins MAIN and MAIL1 interact with PP7L, forming a complex involved in TE silencing through an unknown mechanism. (**E**)**.** SMC4 cooperates with other epigenetic pathways to repress TEs, presumably by promoting high-order chromatin organization. (**F**)**.** MOM1 interacts with PIAL proteins to repress TEs using an elusive process. (**G**)**.** MORC proteins promote constitutive heterochromatin compaction to ensure TE silencing by topological DNA loop trapping mechanism. CC: coiled-coil domain (**H**). The SILENZIO factor interacts with MDB5 and MDB6 and HSP factors to read mCG and silence TEs. Crosstalk and synergistic effects have been described between several epigenetic pathways.

Methylation of the DNA molecule can either occur on adenosines (6mA) or cytosines. Methylation of the 5th carbon of cytosines (5-methylcytosine (5mC)) is a major epigenetic mark conserved in many bacterial, archaeal and most of the eukaryotic genomes, including fungi such as *Neurospora crassa*, plants and most animals [31,32,33]. In plants, 5mC can occur in three cytosine contexts: mCG, mCHG and mCHH (where H = A, T or C), whereas in mammals, it almost exclusively happens in the CG context [31,33]. Together with the histone H3 lysine 9 dimethylation mark (H3K9me2) in plants and histone H3 lysine 9 trimethylation mark (H3K9me3) in animal and fungi, 5mC is strongly enriched at repetitive DNA elements such as TEs that compose constitutive heterochromatic compartments [7,34] (Figure 1A,B). 5mC is established (or de novo 5mC) and then is maintained by specialized enzymes conserved in eukaryotes. De novo 5mC is catalyzed by DNA methyltransferase 3A (DNMT3A) and DNMT3B in animals. In plants, de novo 5mC in all cytosine contexts requires DOMAINS REARRANGED METHYLATRANSFERASE 2 (DRM2) orthologs. DRM2-mediated de novo 5mC requires many plant factors of the so-called RNA-directed DNA methylation (RdDM) pathway, including RNA-DEPENDENT RNA POLYMERASE 2 (RDR2) and DICER-LIKE 3 (DCL3), producing 24-nt siRNAs, ARGONAUTE 4 (AGO4), as well as the plant-specific RNA polymerase IV (RNA Pol IV) and RNA Pol V proteins, acting in concert in transcriptional gene silencing (TGS) (reviewed in [34,35,36]). Maintenance of mCG is catalyzed by DNMT1 and DNA METHYLTRANSFERASE 1 (MET1) in animals and plants, respectively. In *A. thaliana*, depending on the genomic locations, CHG methylation is maintained by CHROMOMETHYLASE 2 (CMT2) and CMT3 through a self-reinforcing feedback loop involving H3K9me2, which is catalyzed by SU(VAR)3-9 homologs (SUVH) 4 (or KRYPTONITE (KYP)), SUVH5 and SUVH6 (Figure 1B) [37]. Finally, maintenance of CHH methylation is assured by DRM2 or CMT2 depending on genomic locations [38,39]. It is therefore important to stress that DRM2 acts either at the initial stage of 5mC (de novo in all cytosine contexts) or at the maintenance step, mostly to preserve 5mC in the CHH context (Figure 1A).

### 2.2. Several DNA Methylation Pathways Cooperate to Silence TEs

The role of 5mC in TE silencing was first demonstrated in maize [40]. More recently, it was confirmed that RdDM and CMT pathways play a crucial role in the silencing of several TE families in this plant species [41]. Studies in *A. thaliana* also showed that several classes of TEs are reactivated in epigenetic mutants showing DNA hypomethylation [42,43,44,45,46] (Figure 1A). Likewise in mammals, TEs are derepressed in *DNMT* mutants [47,48,49].

Although de novo and maintenance of 5mC pathways co-exist throughout the plant life cycle, each pathway plays an important role at specific developmental stages or in response to a specific environmental stimulus that can lead to a burst of TEs. Young TEs, capable of transposition, are rapidly targeted by the RNA-DEPENDENT RNA POLYMERASE 6 (RDR6)-dependent RdDM, or non-canonical RdDM [50,51]. The mechanisms by which TEs are targeted by the non-canonical RdDM have been recently described [52]. TEs harbor a low GC content at the third nucleotide position of codons (GC3) when compared with protein-coding genes. This low GC3 correlates with translation inefficiency, caused by ribosome stalling, which triggers the production of epigenetically activated siRNAs (easiRNAs) by the post-transcriptional gene silencing (PTGS) factors RDR6 and DCL2/4 in cytoplasmic siRNA bodies. The formation of these siRNA bodies depends on liquid–liquid phase separation, mediated by the protein SUPPRESSOR OF GENE SILENCING 3 (SGS3), which interacts with RDR6 [52].

Remarkably, a similar scenario was recently described in tomato, in which young TEs undergoing retrotransposition and increasing in copy number are first targeted by PTGS, then by RdDM, and eventually by the CTM3/KYP and MET1 pathways promoting a robust TGS [53]. Then, the authors proposed that over time, as TEs age, undergoing rearrangements and spontaneous mutations impacting their capability of transposition, they would no longer be targeted by CMT3/KYP and MET1, which would relax TGS, allowing basal TE transcription to reestablish a “secondary” RdDM [53]. At the genome level, these old TEs would serve as a siRNA reservoir to protect the host against reinvasion by TEs with similar sequence identity [53].

A major question is how epigenetic factors cooperate to establish the appropriate pattern of 5mC at TE locations. Previous studies reported an intricate relationship between the RdDM and the 5mC maintenance pathways at the heterochromatic regions. In plants with a decreased 5mC in these regions, an activation of RdDM was observed, suggesting an inhibition of RdDM by heterochromatin in *A. thaliana* [50,51,54,55]. Similar observations were made in maize, tomato and rice, where loss of RdDM was followed by a gain of 5mC in euchromatic regions [56,57,58]. As in *A. thaliana*, it was suggested that, conversely, 5mC inhibited RdDM in constitutive heterochromatin by blocking RNA Pol IV and Pol V activities. Indeed, loss of this 5mC resulted in the spreading of RdDM across the genome, diluting RdDM factors and then compromising their efficiency, which is defined as the dilution model [56,57,58]. Thus, in plants, the symmetric 5mC, i.e., mCG and mCHG, widely present in constitutive heterochromatin, would act as a barrier against the RdDM pathway, to concentrate RdDM factories at boundary regions between euchromatin and constitutive heterochromatin. Further experiments need to be performed to test whether the dilution model holds true or not.

A study in *A. thaliana* investigated the reestablishment of 5mC in the progeny of epigenetic mutants impaired in non-CG methylation [59]. The authors showed that recovery of non-CG methylation depended on the TE genomic context. For most of the TEs, the conservation of mCG within their coding region (TE gene) as well as the presence of the histone variant H2A.W were essential to efficiently regain non-CG methylation in an RdDM-independent manner. Conversely, for a subset of TEs defined as gene-like TE (GLT) genes that had lost CG methylation and swapped H2A.W with H2A.Z, the recovery of non-CG methylation was inefficient [59].

Finally, 5mC dynamic at TEs was investigated in *A. thaliana* shoot apical meristem (SAM), in which TEs are more expressed than in surrounding cells [60]. In SAM stem cells, transient upregulation of TEs correlated with an increase in CHG and a decrease in CHH methylation before flowering. This 5mC signature may reflect some epigenetic reprogramming to initiate the correct 5mC state in male meiocytes, protecting the genome from harmful TE mobilization [60].

### 2.3. Histone Modifications, a Brief Overview

Post-translational modifications (PTMs) of histones affect nucleosome structure, which impacts DNA accessibility to other epigenetic players and transcription factors (TFs) regulating gene expression [61,62,63]. Histone acetylation is an active mark of transcription. Enriched in euchromatin, it is deposited by histone acetyltransferases (HATs) and removed by histone deacetylases (HDACs) [64,65]. Histone methylation can either correlate with transcriptional activation or repression. Di- or trimethylation at lysine 4 of histone H3 (H3K4me2/me3) is an active mark of transcription, whereas in plants, H3K9me2 and H3K27me1 are hallmarks of constitutive heterochromatin that are deposited by SUVH4/5/6 and ARABIDOPSIS TRITHORAX-RELATED PROTEIN 5/6 (ATRX5/6), respectively [66,67]. Finally, the facultative heterochromatin mark H3K27me3 is deposited by the Polycomb repressive complex 2 (PRC2), and is mostly enriched at developmentally or environmentally responsive genes that are transcriptionally repressed [5] (Figure 1B).

### 2.4. A Focus on HDA6-Mediated TE Silencing

Among the *A. thaliana* HDACs, HDA6 is essential for TE silencing, and 5mC hypomethylation was reported in *hda6* mutant [68,69,70] (Figure 1B). Previous studies showed that HDA6 interacts with MET1 as well as with SUVH4, SUVH5 and SUVH6, connecting histone deacetylation with CG methylation maintenance and H3K9me2 in TE silencing [71,72,73]. An interaction was also described with the SWItch/Sucrose Non-Fermentable (SWI/SNF) chromatin-remodeling complex (CRC) subunit SWI3B to repress a subset of TEs [74]. Although these former interactors could not be confirmed, recent HDA6 affinity purification followed by mass spectrometry (AP-MS) analyses highlighted the co-existence of several HDA6 complexes [75,76]. It remains unclear as to what is the composition of the HDA6 complex specifically involved in TE silencing.

### 2.5. Evidences of H3K27me3-Mediated TE Silencing?

Although it is well established that PRC2-mediated H3K27me3 deposition plays an important role in gene silencing [5,77], a direct connection between H3K27me3 and TE silencing is not as clear (Figure 1B). This is most likely because TEs display a high level of 5mC, and H3K27me3 and 5mC are largely antagonistic [78,79]. Thus, in wild type (wt) *A. thaliana*, H3K27me3 is usually excluded from TEs. There is, however, at least one exception with *EVADE* (*EVD*) showing an enrichment of H3K27me3 in wt plants, despite the presence of mCG. To explain this discrepancy, it was proposed that H3K27me3 accumulation at *EVD* was due to the low density of mCG at this location, a feature that was also observed in other species or specific conditions ([80] and see below).

In tissues with low 5mC, like the seed endosperm in plants or during the embryonic development in mammals where DNA demethylation waves are observed, TEs are targeted by H3K27me3 to potentially repress them [81,82]. Furthermore, in DNA hypomethylated *A. thaliana* mutants such as *met1* or *decreased in dna methylation 1 (ddm1)*, an enrichment of H3K27me3 at TEs losing m5C was observed [79,83]. In *ddm1*, this enrichment was dependent on the PRC2-component SET-domain protein CURLY LEAF (CLF) [83]. Remarkably, the *ddm1 clf* double mutant did not show a global increase in TE expression, but instead a partial rescue of *ddm1* silencing defects that is most likely attributed to DNA hypermethylation. Again, *EVD* behaved as an outlier, showing increased transcription and transposition rates in *ddm1 clf* [83]. Therefore, at least for the young TE *EVD*, CLF-mediated H3K27me3 deposition can act as a backup silencing system in *ddm1* mutant plants.

In parallel, a study analyzing *A. thaliana* plants impaired in the two Jumonji H3K27me3 histone demethylases EARLY FLOWERING 6 (ELF6) and RELATIVE OF EARLY FLOWERING 6 (REF6) showed transgenerational epigenetic defects associated with a loss of 5mC and gain of H3K27me3 at heterochromatic TEs [84]. Of note, this gain of H3K27me3 was not sufficient to efficiently repress TEs. Altogether, these studies demonstrate the complex interaction of epigenetic pathways, including PRC2-mediated H3K27me3 to silence TEs. In the future, it will be important to clarify the molecular processes recruiting PRC2 to promote H3K27me3 deposition at TE locations. In mammals, it was shown that *mariner* TEs carry silencer elements that are similar to the Polycomb/Trithorax response elements (PRE/TREs), allowing the recruitment of PRC2 to dampen TE expression [85]. Likewise, some *A. thaliana* TEs carry DNA motifs that are potentially recognized by PRC2 [83]. Furthermore, it was also proposed that H3K27me3 enrichment at TEs could occur by local spreading from nearby genes targeted by PRC2 [80]. To fully unravel the potential role of H3K27me3 in TE silencing, future studies will have to tackle difficult questions such as: what is the connection between H3K27me3 and other epigenetic factors (also see Section 2.6)? Is PRC2-mediated H3K27me3 required for TE silencing? In addition, how is PRC2 recruited to TEs?

In other organisms with a low genomic 5mC level, H3K27me3 enrichment at TEs is also observed: such as in the ciliates *Paramecium tetraurelia* or *Tetrahymena thermophila,* in which PRC2 catalyzes the deposition of both H3K9me3 and H3K27me3 on TEs [86,87], or in the red algae *Cyanidioschizon merolae,* where 50% of TEs are covered by H3K27me3, suggesting that this repressive mark might be involved in TE silencing [88]. Furthermore, H3K27me3 enrichment at TEs was also observed in *Marchantia polymorpha*, a representative species of liverworts, which diverged from other embryophyte lineages in the early Paleozoic, about 500 Mya [89]. Depending on their location in *M. polymorpha* genome, TEs are associated with different epigenetic signatures. While a large proportion of TEs that are mostly located on the highly condensed sex chromosome V are predominantly marked by 5mC, H3K9 methylation and H3K27me1, about 20% of TEs, mostly autosomal, are instead targeted by H3K27me3 and display either a significant reduction or a complete depletion in 5mC level [89]. Thus, targeting of TEs by H3K27me3 would be only possible at genomic locations with low 5mC. The authors propose that TE targeting by H3K27me3 could be explained by an inefficient self-reinforcing loop between 5mC and H3K9 methylation in *M. polymorpha*. Targeting of TEs by H3K27me3 would be an ancient silencing mechanism that would have evolved towards dedicated 5mC- and H3K9-methylation-mediated TE silencing pathways throughout the evolution of other embryophyte lineages (discussed in [90]). In *P. tetraurelia* or *T. thermophila*, loss of function of the PRC2 core subunit Enhancer of Zeste E(z)-like1 (EZL1) induces TE silencing defects [86,87]. Nevertheless, as H3K9 and H3K27 methylation are concomitantly impaired in the *ezl1* mutant, it is difficult to precisely determine the role of H3K27me3 in TE repression. Similarly, whether PRC2-mediated H3K27me3 is required for TE silencing remains to be tested in liverwort.

### 2.6. The Chromatin Remodeler DDM1 Deposits the H2A.W Histone Variant to Silence TEs

Chromatin remodelers are protein factors that catalyze a broad range of reactions impacting chromatin organization and structure, like nucleosome sliding across the DNA or changing the conformation of the histone octamer [91]. All chromatin remodelers use ATP hydrolysis to disrupt the contact between histones and DNA, regulating the access of other factors to the chromatin [92].

The *A. thaliana* AtDDM1 is an SNF2 chromatin remodeler belonging to the mammalian lymphoid-specific helicase (LSH) subfamily that has been studied for decades [93]. This essential epigenetic factor is required for heterochromatin condensation, TE silencing and 5mC maintenance mostly at heterochromatic regions composed of long TEs [39,94,95].

It was initially proposed that DDM1 facilitated DNA methyltransferase activities by evicting the linker histone H1. The *h1* mutant shows a complex 5mC pattern: decrease in CHH methylation at short euchromatic TEs and increased 5mC levels at long heterochromatic TEs [39,96,97]. The effect of *h1* mutation on TE silencing is modest, but in *h1 met1*, mutant synergistic effects can be observed at a subset of TEs showing a substantial upregulation in comparison to respective single mutants [98]. Importantly, H1 subgenomic location does not require DNA methylation or DDM1 activity [98,99]. Instead, AtDDM1 is involved in the deposition of the heterochromatin-specific histone variant H2A.W at long TEs to prevent their mobility in a process that can be dissociated from 5mC and H3K9me2 [99] (Figure 1C). Unlike *ddm1, h2a.w* mutants develop like wt plants, with a minor impact on TE silencing, and a slight decrease of non-CG methylation at pericentromeric TEs [97]. Besides, the H2A.X and replicative H2.A histone variants invade heterochromatin in *h2a.w,* and heterochromatic H1 level is increased. This correlates with a mild reduction in heterochromatin accessibility in *h2a.w* compared to wt plants, a molecular phenotype that is abolished in *h1 h2a.w* double mutant, displaying chromatin accessibility greater than wt plants. It was proposed that H2A.W would regulate H1 occupancy in constitutive heterochromatin by competing with H1 for linker DNA binding in order to optimize heterochromatin accessibility of epigenetic factors such as DNA methyltransferases [97]. Although it is tempting to make connections between these two recent studies [97,99], further analyses will be required to fully apprehend the complex link between these different epigenetic factors. Particularly, the dramatic defects of 5mC or H3K9me2 observed in *ddm1* remain obscure, and cannot be attributed to a loss of H2A.W. One hypothesis is that these epigenetic defects would result from major alterations of the chromatin structure occurring in *ddm1* that would be inherited upon cell division, with concomitantly an inefficient maintenance of 5mC and H3K9me2, together with an invasion of H3K27me3 at heterochromatic TEs.

It is remarkable to note that in humans, the deposition of the heterochromatic histone variant macroH2A by LSH1 is sufficient to repress transcription in a 5mC- and H3K9me3-independent manner [100]. Thus, AtDDM1- and LSH1-mediated histone H2A variant deposition could reflect mechanisms of convergent evolution.

Several studies have investigated the role of DDM1 orthologs in crops, such as tomato, rice or maize. In tomato, disrupting the two *SlDDM1* homologs reallocates the RdDM pathway to heterochromatin, leading to mCHH and siRNA redistribution [57]. In rice, the two OsDDM1 proteins facilitate symmetrical 5mC and H3K9me2 and antagonize RdDM in constitutive heterochromatin [58]. Finally, the maize homologs ZmDDM1a and ZmDDM1b recognize a euchromatic GC-rich DNA sequence, are enriched at transcriptional start site (TSS) of active genes, and by interacting with AGO4 proteins, they would recruit RdDM to promote mCHH in active chromatin [101]. Thus, the connection between DDM1 and RdDM seems to be conserved in crops; whether the euchromatic localization of DDM1 is a general feature in crop species that display a complex genome rich in TEs, remains to be defined. Despite the lack of subgenomic localization or interactome data for *A. thaliana* DDM1, the fact that DDM1 mediates H2A.Z deposition in this model plant will most likely impose the requirement to investigate this aspect for future DDM1-related studies in crops.

### 2.7. The Enigmatic Plant Mobile Domain Proteins

**Figure 2:**
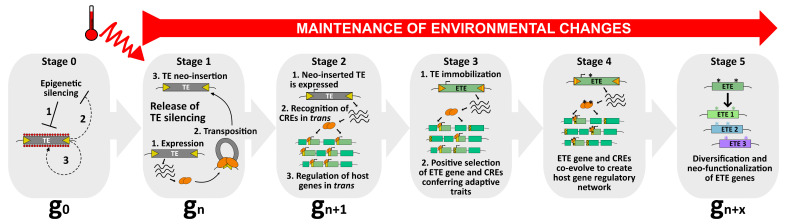
A model to explain the occurrence of TE exaptation. This hypothetical model describes the exaptation of a class II TE-derived transposase. At stage 0, TEs are repressed by silencing pathways to ensure host genome integrity (1). Nevertheless, as purifying selection and genetic drift negatively impact the transgenerational persistence of TEs within the host genome, these latter might use anti-silencing strategies (2) to maintain some basal level of activity (3), and to be inherited as functional elements. Among the pool of TEs, one of them can be considered as an ETE precursor. At stage 1, upon, for instance, the perception of an environmental stimulus, such as heat, this TE is activated. As a young and functional element, it can transpose and mobilize in new genomic regions (neo-insertions). At stage 2, the neo-inserted TE is expressed, producing transposase proteins that will recognize TE-derived CREs genome-wide and potentially recruit additional chromatin factors to regulate host genes in *trans*. At this stage, although the neo-inserted TE is still capable of mobilization, it can enter selective pressure when providing positive advantage to the host. However, this feature must be followed by a swift transition to stage 3, in which the neo-inserted TE becomes immobilized. At stage 3, the neo-inserted TE is immobilized by mutations occurring in its TIRs (red stripes), turning into an ETE gene. Undoubtedly, TE immobilization is essential in the process of TE exaptation. Although TE immobilization is an absolute prerequisite for TE exaptation, it is not sufficient. ETE genes must remain stably expressed, most likely because environmental conditions persist. The ETE gene still undergoes positive selection and CREs that are not conferring adaptive traits are negatively selected. At stage 4, ETE gene and CREs co-evolve, accumulating mutations (*) under purifying selections, and together create a new host gene regulatory network that will be perpetuated only if the environmental changes persist. At stage 5, the ETE gene can be considered a genuine host gene, providing phenotypic value. Following relaxed selection pressure, the TE-derived sequence may inherit point mutations (*), or gene duplication may occur. ETE diversification and neo-functionalization ensure “long-term” positive adaptation of ETE sequence to ultimately create genetic variants with new cellular functions.

The plant mobile domain (PMD) is a protein domain of unknown function only found in angiosperms, and presumably deriving from *Ty3/gypsy* TEs upon TE gene exaptation [102,103,104] (see Section 3 and Figure 2 for the definition of exaptation). The *A. thaliana* PMD proteins were first identified as cellular factors required for SAM and root apical meristem (RAM) organization, genome stability and cell division, hence the name MAINTENANCE OF MERISTEMS (MAIN) for the PMD founding member, and MAIN-LIKE 1 (MAIL1) for its closest homolog [105,106]. Later, it was shown that MAIN and MAIL1 were required for the proper expression of a common subset of genes, as well as TE silencing [104] (Figure 1D). MAIN and MAIL1 physically interact together, as well as with a putative serine/threonine phosphoprotein phosphatase (PPP) called PP7-LIKE (PP7L), and the three mutants show similar developmental and molecular phenotypes, including TE silencing defects [107,108]. Genome-wide level of 5mC is mostly unchanged in *main* and *mail1* mutants, with the notable exception of a slight increase in CHG methylation at pericentromeric regions and in chromosome arm TEs [108]. Importantly, this modest non-CG hypermethylation does not correlate with changes in TE expression observed in the mutant [108]. Synergistic effects were described between MAIN and 5mC pathways. The *main drm1 drm2 cmt3* quadruple mutant showed exacerbation of TE silencing defects, with a number being specifically derepressed in this mutant background [108]. Altogether, these results suggest that together with MAIL1 and PP7L, MAIN would define a distinct epigenetic pathway that cooperates with DRM2- and CMT3-mediated 5mC to silence TEs. The mode of action of PMD MAIN and MAIL1 complex remains largely unknown. Besides, the role of PP7L in this pathway is obscure. As these proteins are at least partially nuclear, determining whether this protein complex can interact with chromatin will be an important question to address.

### 2.8. Structural Maintenance of Chromosome Proteins Get Involved in TE Silencing

Structural maintenance of chromosomes (SMC) proteins compose the core unit of condensin and cohesin ATPase complexes that play an essential role in higher-order chromatin organization, chromosome condensation and sister chromatid cohesion by performing topological entrapment of DNA [109]. The role of SMC proteins in TE silencing was first described in *Drosophila* and *Schizosaccharomyces pombe*, two species largely depleted in 5mC [110,111,112]. In *A. thaliana,* the identification of a hypomorphic *smc4* mutant revealed that SMC4 was required for TE silencing [113] (Figure 1E). In this mutant, several TEs were upregulated, without significant defect in 5mC or siRNA levels. SMC4 can act on strongly methylated TEs, as well as sparsely methylated TEs, demonstrating that 5mC is not required to recruit SMC4 on silenced loci [113]. The authors proposed that conserved histone modifications or histone variants could serve for condensin recruitment, but further investigations are needed to test this hypothesis.

### 2.9. The Elusive MORPHEUS’ MOLECULE 1 (MOM1)

MORPHEUS’ MOLECULE 1 (MOM1) is a plant-specific protein identified in a screen for mutants that released transcriptional gene silencing of a transgenic locus [114] (Figure 1F). MOM1 is required for heterochromatic TE silencing and repression of silent 5S rRNA genes, but *mom1* mutant is not impaired in m5C maintenance or condensation of chromocenters that are interphasic constitutive heterochromatin foci [94]. The protein carries an incomplete ATPase/helicase domain similar to the SWI2/SNF2 chromatin remodeling domain, as well as three motifs called Conserved MOM1 Motif 1 (CMM1), CMM2 and CMM3; CMM2 being the only motif required for MOM1-mediated silencing activity and for its dimerization [115,116]. CMM2 is also essential for MOM1 interaction with PROTEIN INHIBITOR OF ACTIVATED STAT (PIAS)-type SUMO E3 ligase-like 1 (PIAL1) and PIAL2 [117,118]. Besides, MOM1 interacts with small ubiquitin-like modifier 1 (SUMO1) in a non-covalent manner. Although this interaction is not required for MOM1 silencing activity, it was suggested that SUMO1 would play regulatory functions under certain developmental stages or environmental conditions [117]. Although MOM1 was identified 20 years ago, its mode of action remains largely unknown.

### 2.10. The MICRORCHIDIA (MORC) ATPases Repress TEs through DNA Loop-Trapping Mechanism

MICRORCHIDIA (MORC) proteins are gyrase, HSP90, histidine kinase, MutL (GHKL)-type ATPases conserved in prokaryotes and eukaryotes [119,120]. A connection between MORC proteins and TE silencing was first described in *A. thaliana*, through the identification of *atmorc1* and *atmorc6* mutants showing upregulation of several TEs and decondensation of constitutive heterochromatic chromocenters [121] (Figure 1G). The two AtMORC1 and AtMORC6 proteins form heteromers localizing near chromocenters, and it was suggested that MORC ATPases would be involved in higher-order heterochromatin compaction to maintain TE repression [121,122]. In mouse and nematode, MmMORC1 and CeMorc1 orthologs are required for TE and gene silencing [121,123]. Remarkably, MmMORC1 is essential for male germline development and fertility [124]. The mechanisms involving MORC proteins in heterochromatin compaction remained elusive until Kim and colleagues showed that CeMorc1 multimerization allowed efficient DNA binding in a sequence non-specific manner to highly compact chromatin using a DNA loop-trapping mechanism [125] (Figure 1G). Considering the high similarity between eukaryotic MORC proteins, it was suggested that this mechanism of chromatin compaction was conserved among eukaryotic MORCs. This observation made in nematode is consistent with the fact that MORC proteins act in the silencing of a diverse array of sequences in different organisms. Furthermore, it differentiates MORC from condensing complexes that compact DNA by loop-extrusion mechanism [125]. The mechanisms by which *A. thaliana* MORC proteins are recruited to constitutive heterochromatin have been extensively studied. AtMORC6 interacts with several RdDM components, and artificial tethering of AtMORC6 to chromatin is sufficient to recruit the RdDM pathway [126,127,128]. Similar observations were recently made with AtMORC7 [129]. AtMORC7 interacts with AtMORC4 as well as with AtMORC1, AtMORC6 and RdDM factors. Like AtMORC4, AtMORC7 is enriched at RdDM subgenomic sites [129]. From these different studies, it was suggested that AtMORCs and RdDM factors would cooperate to mutually recruit each other; AtMORCs act as tethers promoting efficient RdDM subgenomic retargeting (Figure 1G). Transferring this “MORC tethering” model to mammals could provide some clues regarding the mechanisms involving MmMORC1 in de novo 5mC and TE silencing in mouse male germline [123].

### 2.11. The J-Domain Protein SILENZIO Enters the Game

Lately, a study described the involvement of the two *A. thaliana* methyl-CpG-binding domain proteins MBD5 and MBD6, which are mCG readers, in the subgenomic recruitment of the J-domain protein called SILENZIO (SLN) that acts as a transcriptional repressor [130] (Figure 1H). Transcriptomic analyses revealed that a common subset of TEs was upregulated in *sln* and *mbd5 mbd6* mutants. Moreover, artificial chromatin tethering of SLN was sufficient to promote gene silencing. Besides, SLN, MBD5 and MBD6 interact with several HEAT SHOCK PROTEIN 70 (HSP70) chaperones [130]. This protein complex might potentially recruit unknown transcriptional repressors or inhibit uncharacterized activator to repress TEs in an undetermined mechanism taking place downstream of 5mC.

### 2.12. An Evolutionary Arms Race between TEs and Their Host

The interaction between TEs and their host relies on a perpetual conflict [27]. While the host must dampen TEs because of their harmful effects, using, among others, epigenetic pathways, the TEs must find alternatives in order to perpetuate. Thus, some TEs have developed strategies to overcome the host silencing machinery (Figure 2, stage 0). For instance, upon burst, the evolutionary young *ATCOPIA93* TE called *EVD*, which is initially targeted by the PTGS pathway, uses the host alternative splicing machinery to preferentially synthetize a subgenomic RNA that is dedicated to the production of GAG proteins with protective effects on TE RNAs [131,132]. This allows *EVD* to efficiently multiply in *A. thaliana* genome, until it reaches approximately 40 genomic copies, triggering canonical RdDM and TGS [132]. Another strategy was described for the class II *VANDAL21* TE that encodes the silencing suppressor VANC21, specifically promoting DNA hypomethylation at *VANDAL21* TE sequences [133,134]. Finally, the PMD MAIN and MAIL1 TE silencing effectors are phylogenetically related to TE-encoded proteins, suggesting that TEs may produce their own PMD proteins as a counter-silencing strategy [104,108]. Altogether, these studies illustrate the evolutionary arms race between TEs and their host, relying on a fine balance between TE fitness and host genome integrity.

## 3. From Stress-Induced TE Reactivation to Neo-Insertions with Adaptive Benefits That Fuel Genetic Innovation and Become Exapted

Although primarily considered as junk DNA or parasitic elements, several studies revealed over the years that TEs can be beneficial for their host genome, and therefore are positively selected. There is indeed an accumulation of reports depicting TEs as major drivers of genome adaptation and potential contributors to evolutionary genetic innovation [25,27,135,136].

In natura, environmentally challenged organisms are prone to undergo massive TE reactivation, also known as TE burst, which, upon mobilization, will generate genomic structural variations (SVs) [12,137,138,139,140]. As transcriptional units, TEs carry their own cis-regulatory elements (CREs) that are potentially recognized upon specific stress perceptions. A well-known example is the *A. thaliana ONSEN* LTR retrotransposon that is transcriptionally activated following heat stress due to a heat response element (HRE) located in its LTR [141,142]. *ONSEN* activation is subsequently followed by transposition in several heat-stressed RdDM mutants, demonstrating the importance of this epigenetic pathway to regulate TE mobilization [141]. Although often neutral, TE transposition can promote gene mutations with deleterious effects for the host. Conversely, TE-transposition-induced gene mutations could confer, in some instances, a positive advantage to the host. A study showed that *ONSEN* insertions in two abscisic acid (ABA)-responsive genes improved ABA-related stress tolerance, suggesting TE-driven positive events of adaptation upon environmental changes [143]. Thus, this in-lab study illustrates perfectly how TE-induced SVs can potentially confer a new beneficial adaptive trait (or initial selective advantage) that might engage in natural selection as long as its benefit to the host is maintained. Remarkably, this is fully consistent with the precursory McClintock’s theory foreseeing TEs as potential important drivers of genome reorganization in response to environmental “shocks” [144].

TE-induced SVs have been well documented during the process of plant domestication, in which these events are selected and maintained under high selective pressure by humans, as they provide selective advantages in relation to desired agronomic traits ([23,145,146] and see Section 5.1). TE insertion polymorphisms have been described in several crops, including rice, in which it is an ongoing process still occurring in the field [147]. It is noteworthy to mention that plant domestication is often associated with polyploidization through whole genome duplication (WGD) [148]. Considering that TE mobilization is often observed during WGD [149], that would explain why plant domestication and TE mobilization are genetically connected.

At the macroevolution scale, the phenomenon leading to the appropriation of TE-derived sequences by the host is defined as TE exaptation—also known as co-option or TE domestication [26]. Exaptation is an evolutionary term in the taxonomy of fitness that was initially introduced by Gould and Vbra [150]. The authors claimed that the concept of adaptation, in which natural selection shapes a specific character for its current use, was not sufficient to explain all morphological innovations. They proposed two other processes, together coined exaptation. In the first process, a character whose function was originally shaped by natural selection (an adaptation event) is reallocated to a new function (co-option). In the second, the origin of a character that was subsequently co-opted for its current use cannot be explained by natural selection (a “nonaptation” event) [150]. As a long-term evolutionary process, TE exaptation is the outcome of an initial TE neo-insertion event, most likely occurring swiftly, and conferring a selective advantage to the host at a given time. Under constant environmental constraints, this neo-inserted TE will undergo positive selection to eventually become, in a long timescale, an exapted TE (ETE) gene or exapted TE-derived sequence (Figure 2).

**Figure 3:**
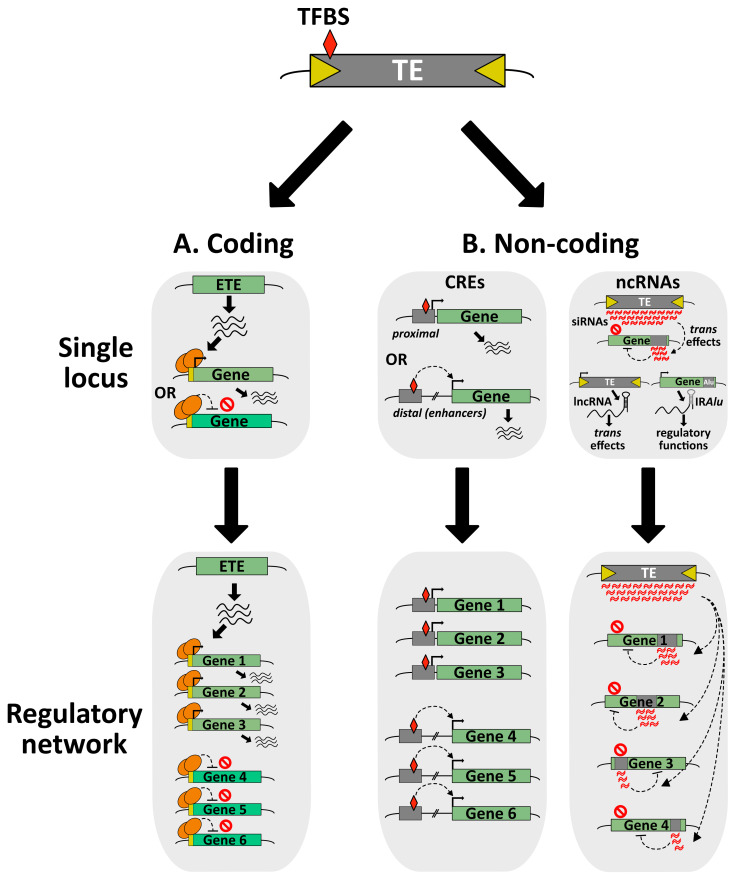
From single locus positive selection of TE-derived sequences to the elaboration of complex regulatory networks. (**A**) Co-option of a TE gene can positively impact the host through the regulation of specific gene with the ETE proteins acting as transcriptional activators or repressors, most likely by recruiting other chromatin factors (not represented here). At the genomic scale, ETE proteins can participate in large regulatory networks ensuring the fine tuning of gene expression. (**B**) Co-option of non-coding TE sequences acting as CREs can regulate the expression of single locus through proximal or distal interactions with the transcriptional machinery. Genome-wide, these TE-derived CREs can be part of interconnected gene regulatory networks. TFBS: transcription factor (TF)-binding sites. Co-option of non-coding TE sequences acting as ncRNAs can either target a discrete host gene as siRNAs or produce lncRNAs with *trans* regulatory functions. In the case of *SINE Alu* elements located in the 3′UTR of host genes, they can form IR*Alu* secondary structure of mRNAs involved in splicing, mRNA subcellular localization and other processes. In a hypothetical model, TE-derived siRNAs can engage in complex regulatory networks repressing several host genes.

At the molecular level, TE exaptation can be of various natures, through the co-option of coding or non-coding (nc)DNA/RNA sequences (from TE genes or TE-derived regulatory elements) (Figure 3). Nowadays, it is well-accepted that TE exaptation has been a major driver for several genetic and morphological innovations throughout evolution [25,135]. There are many examples depicting TE exaptation events as major contributors to essential evolutionary transitions, such as the elaboration of the vertebrate adaptive immune system [151,152,153], or the development of the embryo-nourishing tissues that are the mammalian placenta and the seed endosperm [154,155].

## 4. Exaptation of TE Coding Regions

One of the most striking beneficial contributions of TEs to the host genome is illustrated by the exaptation of TE-derived protein-coding sequences, which is a phenomenon leading to new functional host genes that can be referred to as ETE genes. It is in *Drosophila* in 1992 that Miller and colleagues identified the first P element-derived ETE genes; the authors suggested that TE exaptation was an evolutionary process potentially favoring genetic innovation and creating new biological functions for the host organism [156]. Since then, ETE genes have been discovered in numerous organisms: from unicellular organisms like prokaryotic archaea and bacteria, yeast and ciliates to multicellular organisms such as insects, metazoans and plants [25]. Considering that TEs compose the genomes of virtually all living organisms, it is tempting to speculate that ETE genes are present in any form of life.

TEs have diverse, attractive and sophisticated molecular tools, making them a dynamic reservoir for potential ETE genes with new cellular functions. Their genomes encode transposases, integrases and reverse transcriptases, as well as structural and envelope proteins that can be co-opted by the host during evolution [25]. Notably, the elaboration of a new ETE gene can occur through the co-option of a whole TE gene or a fragment of it. In addition, ETE gene or fragment can be fused to a preexisting host gene, creating a new chimeric protein. Although there are several reports of class I TE-derived ETE proteins, the majority of ETE proteins are highly similar to class II TE transposases [25,157]. This discrepancy between class I and class II TEs as reservoir of ETE genes can be explained either by an easier identification of class-II-derived ETE genes, or by the fact that there is a real preference for transposase-derived ETEs. Transposases have two essential domains: a specific DNA-binding domain (DBD) and a catalytic DDE or DDD endonuclease domain responsible for DNA cleavage and transposon integration. Although a large number of the ETE genes encode the two domains, several ETE proteins only carry the DBD. This may explain why several ETE proteins are presumably acting as transcription factors (TFs), either activators or repressors of transcription (Figure 3A). Besides, ETE proteins are involved in various chromatin-related processes such as TE silencing, mRNA splicing, DNA repair, telomere integrity, centromere formation, chromosome segregation and recombination, or in other cellular processes regulating translation or nuclear import [15,25,157]. To date, there are more than one hundred and fifty identified eukaryotic ETE genes. For most of them, the biological role and molecular mechanisms remain unknown [15,25,157,158].

### 4.1. The FAR1/FH3 TF Family: Multitool ETE Proteins Involved in Environment Sensing and Plant Development

In plants, the first and by far the most thoroughly characterized ETE genes are *FAR-RED IMPAIRED RESPONSE1 (FAR1*) and *FAR-RED ELONGATED HYPOCOTYLS3 (FHY3)*. FAR1, FHY3 and homologs are TFs deriving from *Mutator*-like (MULE) transposases [159]. In *A. thaliana*, FAR1 and FHY3 act cooperatively downstream of the photoreceptor phytochrome A to specifically modulate far-red light-responsive gene expression [160]. These proteins are also required for various processes such as chlorophyll biosynthesis, circadian rhythm, shade tolerance, seed germination, flowering, plant immunity and stress responses [161,162,163,164,165,166,167]. Through their DBD, FHY3/FAR1 complexes recognize specific CREs, called FHY3/FAR1-binding site (FBS) that are predominantly located at the transcription start site of promoters [160]. FBS are enriched in the promoters of numerous genes exhibiting diurnal or circadian cycling, like for instance, *CIRCADIAN CLOCK ASSOCIATED1* (CCA1), which is a central gene controlling the circadian clock [160,166]. Conversely, Xie and colleagues reported a new function of FHY3 and FAR1, acting as transcriptional repressors. They found that the FHY3/FAR1 complex physically interacts with three SQUAMOSA-PROMOTER BINDING PROTEIN-LIKE (SPL) TFs. This complex negatively regulates the binding of SPL to the DNA at the promoter of several genes involved in flowering regulation, including FRUITFUL (FUL), LEAFY (LFY) and APETALA1 (AP1). Thus, FHY3/FAR1 complex induces the downregulation of key flowering time master regulators, which ultimately delays flowering [168]. Altogether, these studies demonstrate that FAR1/FHY3 and their homologs are major TFs involved in the perception of environmental changes during plant development.

There are fourteen genes belonging to the FAR1/FHY3 family in *A. thaliana* [169]. FAR1/FHY3-related TFs are conserved in other eudicot species, as well as in monocots, suggesting that exaptation of these sequences occurred prior to the monocot–dicot split [170]. Comparative genomic analyses have revealed large proportions of *FAR1/FHY3* genes in several plant species. A significant expansion of an *FAR1/FHY3* gene family would correlate with the prostrate-to-erect growth switch specifically observed in the domesticated *Rosa wichuraiana* “Basye’s Thornless” (BT) species, with potential links to molecular processes related to light signaling, shade perception and flowering time [171]. The large diversification of *FAR1/FHY3* genes observed in the wild species *Medicago ruthenica*, in comparison to two related species, *M. truncatula* and *M. sativa*, could contribute to the higher tolerance of *M. ruthenica* to various abiotic stresses including drought, making it a valuable alternative as a legume forage crop [172]. In the tea plant (*Camellia sinensis*), the steady-state level of *FAR1/FHY3* mRNAs is highly modulated in response to various abiotic stresses such as high salt, high/low temperature, polyethylene glycol (PEG)-mimicked drought or abscisic acid (ABA) treatment [173]. Altogether, these reports are consistent with a previous study performed in *A. thaliana* showing that mutant plants knocked out for genes belonging to the *FAR1/FHY3* family display changes of their phenotypical traits when challenged by several abiotic stresses [174]. Thus, FAR1/FHY3 TFs can be depicted as multifaceted molecular players involved in plant development and in response to various environmental stimuli.

### 4.2. The hAT-Derived SLEEPER Genes

*DAYSLEEPER*, the founding member of the *SLEEPER* gene family, was first discovered in a yeast one-hybrid screen aimed at identifying *Arabidopsis* proteins bound to the promoter of *Ku70*, a gene involved in DNA repair, through the recognition of the DNA Kubox1 motif [175]. *DAYSLEEPER* encodes a protein carrying the canonical transposase domains of *h*AT DNA transposons (*hobo* from *Drosophila* or *hermes* from housefly, *Activator* from maize and *Tam3* from snapdragon) that are a multiblock hAT dimerization domain required for self-interaction, and a BED-type ZF domain interacting with DNA [176]. However, the protein lacks the catalytical DDE motif typical of *h*AT transposases [177]. *DAYSLEEPER* has also lost the flanking 8-bp genomic target-site duplications (TSDs), hallmarks of *h*AT integration, and the terminal inverted repeats (TIRs) that are required for transposition [177]. While *daysleeper* null mutants showed dramatic developmental phenotype, failing to develop normal organs, plants overexpressing *DAYSLEEPER* were capable of developing despite morphological defects [175]. Gene expression profiling of *DAYSLEEPER*-overexpressing plants revealed several upregulated genes, although none of them carried a Kubox1 motif in their promoter. *DAYSLEEPER* is highly expressed in SAM and RAM, reproductive organs and seeds [175]. Subcellular localization experiments confirmed that GFP-tagged DAYSLEEPER localized into the nucleus, which is consistent with the molecular function of the protein and the presence of a nuclear localization signal (NLS) [176]. Besides, overexpressing fluorescent protein (FP)-tagged DAYSLEEPER in *Arabidopsis* protoplasts revealed a cytoplasmic localization in multi-vesicular bodies and potentially late endosomes [176]. Considering that FP-DAYSLEEPER proteins were overexpressed, further studies will be required to determine the biological significance of its cytoplasmic localization.

In *A. thaliana,* the *DAYSLEEPER* closest homolog was called *CYTOSLEEPER* because the protein is cytoplasmic, which is consistent with the absence of an NLS. Unlike *DAYSLEEPER* loss-of-function, *cytosleeper* mutants are undistinguishable from wt plants [178]. Seeking for *SLEEPER*-like genes using the conserved “sleeperdomains” identified several candidates in the angiosperm sister species *Amborella trichopoda* as well as in distant species such as the water lily *Nuphar variegata.* In *Oryza sativa*, several *SLEEPER*-like genes were identified and named *RICESLEEPERs.* Remarkably, functional studies carried out in rice strongly suggested that *RICESLEEPER1* and *RICESLEEPER2* genes were both crucial for plant growth, as only heterozygote mutant plants could be retrieved [178]. Altogether, these studies demonstrated the pivotal role of *DAYSLEEPER* and relatives in plants.

### 4.3. The Angiosperm-Specific MUSTANG Family

Unlike the *FAR1/FHY3* or *DAYSLEEPER* genes that were identified by classical genetic approaches, the *MUSTANG (MUG)* genes were discovered by in silico analyses seeking for MULEs TE-derived *mudrA*-like ETE genes conserved in several angiosperms including *A. thaliana* and *O. sativa* [179]. The *A. thaliana* genome encodes eight MUG homologs gathering into two distinct clades: MUGA (MUG1 to MUG4) and MUGB (MUG5 to MUG8) [180]. It has been suggested that *MUGA* and *MUGB* would have arisen from two independent exaptation events [170]. *MUG* genes act redundantly to ensure proper plant development. Although *mug1, mug2* and *mug3* simple mutants are phenotypically undistinguishable from wt plants, high-order mutant combinations such as *mug1 mug2* or *mug1 mug2 mug3* can seriously impact plant growth, flower development and fertility [170,180]. Similarly, the *mug7 mug8* double mutant displays severe growth delays [180]. Like FAR1, MUG proteins carry the typical MULE transposase domain and a divergent SWIM ZF. They also carry an MuDR domain. In addition, the MUGB MUG7 and MUG8 carry the Phox and Bem1p (PB1) domain of unknown function, most likely acquired through a transduplication event (see Section 4.6). It was proposed that MUG proteins would contact DNA with their MuDR domain and/or SWIM ZF, acting as putative transcriptional regulators of plant development [180]. As observed with *far1/fhy3* mutants, phenomic studies showed that the growth of several *mug* mutants is impacted by phosphate starvation or various abiotic stresses [174].

### 4.4. The PIF/Harbinger-Related ETE Proteins

Originally identified in maize but also present in animals, the *P instability factor (PIF)/Harbinger* superfamily is composed of class II TIR DNA TEs encoding two distinct proteins: a superfamily-specific transposase with a DDE triad for endonuclease activity, and a DBD protein with a conserved SANT/myb/trihelix motif [152]. The first example of *PIF/Harbinger*-derived ETE gene was *Harbinger Transposase-Derived 1* (*HARB1)*, identified in humans and several mammals, chicken, frogs and fish [152]. Remarkably, exaptation of *Harbinger*-derived transposase and DBD proteins occurs concomitantly, in pairs. Thus, HARB1 was found to interact with the co-opted DBD protein nuclear apoptosis-inducing factor 1 (NAIF1), and functional homologies in the molecular mechanisms involving *Harbinger* transposase/DBD proteins and their domesticated counterparts have been proposed [181]. To date, the function of the HARB1/NAIF1 remains unknown.

Exaptation of *PIF/Harbinger* TEs is frequent in *Drosophila* [182], and HARB1-like proteins have been identified in plants. In *Malus domestica* (apple), *MdHARB1* transcription is induced by heat stress, and transgenic plants overexpressing *MdHARB1* are more resistant to heat [183].

In *A. thaliana*, several co-opted *Harbinger* proteins have been related to various epigenetic pathways. The tendency of *Harbinger* TEs to be inserted in gene-rich regions of the genome, as well as the dual nature of their transposase module could have predisposed them to exaptation as components of chromatin-related processes. Besides, considering that epigenetic pathways repress TEs, it was proposed that co-option of TE genes to be reallocated to these cellular processes could reflect an adaptation to evolutionary conflicts in the never-ending fight between the host and invasive elements [25].

The transposase ANTAGONIST OF LIKE HETEROCHROMATIN PROTEIN 1 (ALP1) and the divergent DBD protein ALP2 interact together to antagonize PRC2-mediated H3K27me3 deposition and gene silencing [184,185]. Importantly, ALP2 is required for the interaction between ALP1 and MULTICOPY SUPPRESSOR OF IRA1 (MSI1), a core component of PRC2 [185]. ALP1/ALP2 subgenomic locations are unknown; more experiments and structural approaches of higher resolution are needed to unravel the significance of the various protein domains in the ALP1-ALP2-MSI1 interaction. Besides, the effect of *alp1* and *alp2* mutants on TE silencing is unknown. Nevertheless, these functional studies clearly showed the importance of ALP1 and ALP2, most likely by controlling PRC2 activities during important developmental transitions.

Another example of dual exaptation involves the transposase Harbinger-derived protein 1 (HDP1) and the DBD protein HDP2. The two proteins interact together as well as with several components of the increased DNA methylation 1 (IDM1) histone acetyltransferase complex, including the methyl-DNA-binding protein MBD7, to prevent DNA hypermethylation and TE silencing [186]. HDP2 DBD is capable of binding DNA in vitro, and at the genomic level, the protein is significantly enriched at MBD7 locations [186]. Thus, the co-opted HDP1 and HDP2 are recruited to histone acetyltransferase complex to promote basal TE expression and antagonize DNA methylation-mediated gene silencing. It will be important in the future to precisely delineate the biological significance of this phenomenon.

Finally, the co-opted transposase HDA6-associated Harbinger transposon-derived protein 1 (HHP1) was recently identified as a new HDA6 partner, forming a complex together with the four Harbinger-derived SANT-domain-containing proteins SANT1, SANT2, SANT3 and SANT4, and the MBD1, MBD2 and MBD4 [76]. Then, split luciferase assays suggested that HHP1 and MBD1 would act as bridges between HDA6 and the SANTs. Although the *hhp1* or *mbd1/2/4* mutants did not show abnormal growth, the *sant* quadruple mutant displayed a late flowering phenotype similar to the *hda6* mutant. Further analyses revealed that SANT and HDA6 co-regulate histone acetylation and are required for the proper expression of a common subset of genes, such as the floral repressor *FLOWERING LOCUS C*. It is unknown whether HDA6 targeting at these misregulated genes required the DBD of SANT proteins. Besides, it will be important to precisely delineate the role of HHP1 and MBDs in this pathway. Considering that these proteins are most likely required for the indirect interaction of HDA6 and SANT proteins, it would be relevant to test whether the *hhp1 mbd1/2/4* quadruple mutant displays a late flowering phenotype.

### 4.5. TE Exonization and Exon Shuffling to Create New Chimeric Proteins (Host Gene-TE Fusion)

TE exonization is a molecular process leading to the creation of new exons from mutated introns composed of TE sequences [158]. TE exonization is especially prevalent in animals, where alternative splicing is largely observed [187]. Exon shuffling promotes the rearrangement of exons from different genes to elaborate new chimeric genes, a process that can be sourced from TEs [188]. Importantly, TE-mediated exon shuffling can solely involve host gene sequences (also see Section 4.6), or integrate TE sequences in the process. Together with TE exonization, TE-mediated exon shuffling is an important molecular process contributing to innovations of the host genome by creating new cellular functions through genetic rearrangement [15,157,158].

A striking example of host gene-transposase fusion (HTF) is the primate gene *SETMAR* which is composed of a *Tcl-Mariner* transposase inserted downstream of a *SET* histone methyl transferase gene [189,190]. The SETMAR DBD has kept its specificity for a DNA motif derived from the ancestral *Tcl-Mariner* terminal sequence (Hsmar1 ITRs) that is still present in thousands of copies in the human genome. A modest overexpression of SETMAR leads to the misregulation of 1500 genes, with an enrichment for the Hsmar1 ITRs in upregulated loci. Furthermore, these genes may be involved in cancer [191]. The exaptation of *SETMAR* gene occurred in the anthropoid primate lineage. The three *SETMAR* protein domains, i.e., its SET, catalytic transposase and DNA-binding domains are under a strong purifying selection [192]. All these results suggest that the fusion of the ancestral transposase and SET genes in anthropoid primates would have contributed to the emergence of new gene regulatory networks.

Recently, an elegant analysis using comparative genomics surveyed the occurrence of HTFs in available tetrapod genomes [193]. The authors identified 106 HTFs deriving from 94 independent fusion events over the course of ~300 million years of evolution. Analysis of *HTF* gene structure revealed that transposase capture occurred through alternative splicing events. All known eukaryotic DNA transposon superfamilies contribute to HTF formation, with a predominance of Tc1/mariner-, hAT- and P element/Kolobok-derived transposases fused to host domains involved in transcriptional regulation. While host gene/transposase-derived DBD fusions are prevalent, HTFs can also involve whole transposase sequences. For instance, the vesper bats KRABINER protein derives from an HTF event between the host transcriptional repressor Krüppel- associated box (KRAB) and a full-length *Mlmar1* mariner transposase. KRABINER is involved in transcriptional repression in a DNA-sequence-specific manner, and binds genome-wide hundreds of cognate TE-derived CREs, controlling a large network of genes [193]. The fact that several HTFs are composed of host domains connected to chromatin, such as KRAB, SCAN zinc finger (ZF) or SET domains, suggests that most of the HTF proteins would play a role in chromatin-related processes, possibly acting as activator or repressor TFs [193].

In humans, intronic SINE *Alu* elements are particularly prone to TE exonization, through the use of cryptic splice sites residing within their sequences [194,195]. Thousands of human gene transcripts may contain TE-derived sequences [196,197,198]. Recently, it was shown that an alternative spliced transcript (CD274-L2A) was generated by exaptation of an intronic LINE endogenous retroelement and created a new variant of PD-L1 (a membrane-bound protein) without the transmembrane domain and the regulatory sequence in the 3′UTR [199].

Advances in sequencing technologies have made it possible to investigate the transcriptional landscape of TEs at the genomic scale. Five prime cap (5ʹ-Cap) capture coupled to nanopore-based long-read sequencing of mRNA was carried out in locust that has a complex genome highly enriched in TEs, to characterize full-length transcripts in their native form and evaluate the propensity for TE exonization. Among the 60,908 representative RNA transcripts, 51.88% of them contained at least one TE-derived sequence, mostly in the first or last exons, but to a lesser extent, in internal exons as well [200]. It will be relevant to develop similar analyses in *A. thaliana* and plants with more complex genomes.

In angiosperms, there are a substantial proportion of genes carrying TE fragments. For example, in rice, more than 10% of transcripts were reported to contain TEs or TE-derived sequences, and TEs contribute to about 2% of rice protein coding regions [201]. Similarly, in *A. thaliana*, 7.8% of expressed genes were found to contain a region with close similarity to a known TE sequence [202]. Recently, in mulberry, it was shown that the proportion of MITE-associated genes was about 1,5%, and the proportion of MITE-related alternative splicing was 2,5% to 5% compared to the number of all alternative splicing events, according to the tissues tested, bud and flower, respectively [203]. This illustrates the great potential of TE-driven genetic diversification through alternative splicing events.

### 4.6. Transduction and Transduplication

As mentioned above, TEs have a propensity to capture and transpose entire host genes or fragments through the process of TE-mediated exon shuffling, which can result in gene duplication or rearrangement. This phenomenon is named transduction or transduplication, depending on the involvement of class I or class II TEs, respectively [204]. Although the molecular mechanisms and evolutionary consequences of these phenomena for the host as well as for TE fitness are not well understood, there is an increasing amount of evidence proving the importance of these processes in the elaboration of genetic diversity.

Class I TE-mediated transduction involves transcriptional readthrough from the retrotransposon promoter to the adjacent host gene sequence and its subsequent incorporation into the TE sequence during reverse transcription [205]. There are few cases of transduction in plants. In maize, transduction involving the LTR retrotransposon *Bs1* created a novel chimeric gene composed of three host gene fragments fused to the *Bs1* gag domain. This chimeric gene is expressed at the protein level, and its function could be involved in reproductive development [206]. Another example is *Katydid-At1*, an element of the terminal-repeat retrotransposons in miniature (TRIM) family of non-autonomous LTR retrotransposons that was involved in the transduction of a gene potentially involved in nonsense-mediated mRNA decay (NMD) [207].

In humans, LINE-1 *L1* and *SVA* (SINE/VNTR/Alu) elements also contribute to transduction events of adjacent host sequences [208,209]. It was proposed that mammalian *L1* TEs would be involved in the generation of ten thousand genes through the process of transduction, many of which are transcribed and some of them acquired new cellular functions [210].

In plants, Class II TE-mediated transduplication involves *Pack-TYPE* TEs that are predominantly derived from MULE, CACTA and Harbinger TEs in *A. thaliana*. *Pack*-TEs share many features with host genes. They are usually not highly repeated, and enriched in euchromatic instead of pericentromeric regions. Pack-TE-mediated transduplication events generate genes that are under purifying selection [211,212]. This implies that transduplication events may have a direct influence on genome evolution, generating new gene functions or regulatory activities by shuffling fragments of various genes across the genome. Zhao and colleagues reviewed very well the features of transduplicated genes in comparison with the original versions or donor genes [164]. The first feature is the size of transduplicated genes, which is usually smaller than primary genes. It is relatively rare for a *Pack*-TE to transduplicate a whole gene. The second feature is that, unlike regular duplicated genes, the alignment of transduplicated gene fragments with their original counterpart reveals a sharp boundary at the breakpoint of duplication. The third feature is that transduplicated gene fragments are often flanked by inverted or direct repeats. The presence of TSD flanking the TE DNA repeats is the unambiguous evidence that the entire structure belongs to a single TE. Furthermore, each transduplication event should be associated with specific flanking TSD. However, TSD are not always trivial to spot, making the identification of this phenomenon difficult. Thus, it is probable that the number of transduplicated genes may be strongly underestimated, as it is only recently that transduplication events may have been identified with confidence. As a consequence, it remains a great challenge to identify this type of gene [164].

As mentioned above, the MUGB MUG7 and MUG8 proteins carry a PB1 domain of unknown function, usually not associated with TEs, that was likely acquired via transduplication before the exaptation of MUGB TE precursor [170,180]. The PB1 domain belongs to an additional short 5′-exon, which is consistent with the general pattern of Pack-MULE transduplication. This phenomenon is interesting because it suggests that the MUGB PB1 domain may have undergone a complete co-evolutionary cycle.

Recently, the first report of a real-time mobilization of non-autonomous Pack-CACTA has been highlighted [213]. In this study, new transposed copies were found mostly within euchromatic regions (65%), which is consistent with previous observations [214]. The authors proposed a model where acquisition of host DNA was tightly linked to transposition, and importantly, DNA methylation played a direct role in controlling the transposition of Pack-TYPE TEs [213]. When two Pack-CACTA are close together, frequent aberrant excisions of one of the two TIRs in each pair allow the remaining termini of the neighboring transposons to encompass a new single element incorporating the host DNA [213].

Finally, the epigenetic state of over a thousand transduplicated genes was analyzed in the maize genome [215]. Remarkably, the level of DNA methylation and siRNAs was higher at donor genes than at genes with no evidence of transduplication capture. Moreover, transduplicated genes were highly DNA methylated and less expressed than donor genes. Furthermore, Pack-TEs mapped fewer siRNAs and were slightly less methylated than related TEs without captured fragments. All together, these observations could reflect some epigenetic conflicts between Pack-TEs and their relative, as well as between donor and transduplicated genes [215]. This undoubtedly depicts the complex interaction between TEs and host genomes.

## 5. Exaptation of TE-Derived Non-Coding Regions

### 5.1. From Positive Selection to Exaptation of TE-Derived Non-Coding Regions

TE mobilization can potentially lead to the production of TE-derived non-coding (nc)DNA sequences with positive selection signatures for host adaptation. They can be TE-promoter-derived CREs used as alternative promoters, as well as enhancers, insulators or repressor DNA elements regulating the transcription of host genes by the recognition of specific TFs [139]. Because of their self-replicative properties, TE-derived CREs can colonize the host genome, making them a powerful driving force to create transcriptional regulatory networks, which under constant environmental constraints, might become exapted (Figure 2). Nevertheless, it is important to mention that defining TE-derived ncDNAs as a genuine exaptation event should always be cautiously assessed [216].

TEs do not mobilize randomly in the genomes, and genomic distribution varies according to TE families, as exemplified in *A. thaliana* [13,217]. Furthermore, some specific TE families tend to preferentially mobilize upstream of genes, in the promoter regions, which is an ideal location to be potentially positively selected as new CREs of adjacent genes [23]. In humans, 25% of promoters and CREs may derive from TEs [218,219]. Comparative analyses of *A. thaliana* and *A. lyrata*, two sister species that diverged 10 million years ago, showed that 16% and 24% of genes harbor a TE insertion in a 500 bp window of the coding region [220]. This difference could be explained by a higher TE content in *A. lyrata* genome [220]. In rice and in maize, the MITE *miniature mPing* and *MuDr* TEs, respectively, preferentially insert into the promoters of genes, which can potentially upregulate adjacent genes [221,222]. In humans, statistical analyses of class I TE sequences for their propensity to harbor TF binding sites (TFBS) revealed that LTRs are most likely to contain almost all known TFBS in comparison to randomly generated sequences [223]. These results clearly indicate that TEs are well equipped to contribute to host gene regulation [224]. It is important to mention that following insertion, class I TEs often undergo ectopic recombination between their LTRs, resulting in the removal of their coding regions, with remnant solo-LTRs. This process allows the dissemination of several hundred thousand solo-LTRs throughout the human genome [225]. Likewise, in rice, 14,7% of LTR-retrotransposons are in the form of solo-LTR [226]. As these solo-LTRs have potentially kept their TF-specific CREs, they can influence the expression of neighbor genes.

Specific epigenetic chromatin states are most likely important factors influencing TE neo-insertions. In *A. thaliana,* while the neo-insertions of *VANDAL21, ATENSPM3* and *ATCOPIA93 (EVD)* TEs occur preferentially in or nearby genes, only *VANDAL21* TEs are significantly more often inserted in the promoters or 5′UTRs of transcriptionally active genes, enriched in H3K4me3 and H3K36me3 active marks. Conversely, *ATENSPM3* and *ATCOPIA93* TEs are more often inserted throughout the whole body of genes that are transcriptionally repressed by H3K27me3 deposition, and enriched in H2A.Z variant [227]. According to Wells and Feschotte, the propensity of some TE families to specifically target promoters of transcriptionally active genes could be a strategy favoring subsequent TE expression and transposition [228]. It is unclear whether the expression levels of neo-inserted TEs correlate with the chromatin state of insertion sites. For instance, in *A. thaliana,* it would be interesting to determine if *VANDAL21* neo-inserted TEs harbor active epigenetic mark and tend to be more expressed in comparison to their *ATENSPM3* and *ATCOPIA93* counterparts, which would display a developmental-specific expression pattern, as they are inserted in or nearby PcG-repressed genes. In a similar approach, Zhang and colleagues analyzed the genome-wide distribution and chromatin landscape of TE neo-insertions in several plant and animal genomes [229]. They noticed that in addition to passive mobilization into open chromatin, several TE families tend to mobilize at TSS and/or transcriptional termination sites (TTS) of actively transcribed genes, suggesting a process of convergent evolution among eukaryotic TE families [229].

As TE promoters often carry stress-responsive elements, TE-derived ncDNAs might to some extent be positively selected to temporally regulate the expression of adjacent genes upon environmental stimuli or stresses [230]. In plants, there are several examples of TE-derived ncDNAs acting as cryptic promoters of nearby genes. One of the most striking examples is in *Citrus sinensis* (orange), where the LTR-retrotransposon *Rider* controls the expression of *Ruby*, a gene encoding a MYB TF required for anthocyanin production. Remarkably, different fruit pigmentations among orange varieties correlate with allelic variation of the cold-responsive *Ruby* locus carrying or not *Rider* in its promoter [231]. As this *Rider* insertion is recent, it should not be considered a TE exaptation. It could, however, be seen as a preliminary step to a future TE exaptation event. Similar cases have been highlighted in other crops, as in maize and apple [232,233]. In *A. thaliana* as well, it was suggested that an unmethylated *ATCOPIA93*-derived solo-LTR had been evolutionarily co-opted to cis-regulate the disease resistance gene *RPP4,* conferring plant immunity advantage [80]. In addition, a study described a LINE TE called *EPCOT3* that was positively selected in *A. thaliana*, but not in *A. lyrata*, to regulate the expression of *CYP82C2* through the binding of WRKY33, a pathogen-responsive TF [234]. The authors showed that *CYP82C2*, which is a gene involved in the production of metabolites for pathogen defense, originated from the duplication of the iron-stress response gene *CYP82C4* in the two *Arabidopsis* species. They proposed that *CYP82C2* underwent neo-functionalization upon *EPCOT3* insertion in its promoter, and subsequent rewiring of WRKY33 regulon, leading to a positive effect on fitness [234]. Although the molecular mechanisms regulating *A. lyrata CYP82C2* transcription remain elusive, purifying selection analyses suggest that it might be involved in pathogen defense.

### 5.2. TE-Derived CREs Shape Transcriptional Regulatory Networks

During transposition bursts and subsequent genomic rearrangements, TEs can spread their own promoter-borne CREs, as well as host CREs through the processes of transduction or transduplication (as described in Section 4.6). When inserted near genes, these TE neo-insertions can ultimately create new transcriptional regulatory networks or rewire existing networks, giving rise to new phenotypes and creating diversity. Furthermore, TEs are sources of lineage-specific regulatory elements, since TEs evolve rapidly, both in sequences and genomic distribution [220,235]. Here, we present examples of transcriptional regulatory networks shaped by TE-derived CREs in several evolutionary lineages that coordinate the expression of host genes involved in complex biological processes (reviewed in [18,139,155,224]).

One of the most outstanding examples of TE-derived CRE network is the elaboration of the mammalian placenta, involving *MER20* DNA transposons in an evolutionary process that occurred more than 100 million years ago [236]. More recently, in the past 15–25 million years, the *RLTR13* endogenous retroviruses (ERVs) rewired hundreds of placenta-specific enhancers in mice, suggesting a role for ERVs in the swift evolution of placenta morphology between mammal lineages [237]. It is noteworthy to mention that ERVs can be structurally indistinguishable from retrotransposons and may represent evolutionary intermediates between these latter and classical retroviruses [238]. Other studies reported TE-based rewiring of cis-regulatory networks in several developmental and cellular processes such as stem cell pluripotency, neo-cortex development, innate immunity and mammary gland evolution [136,239]. The regulatory functions of TEs may differ between cell types and developmental stages. Indeed, TEs can function in host gene regulation as alternative promoters, enhancers or boundary elements [235].

In plants as well, several examples of TE-driven *CRE* networks promoting TFBS rewiring have been described [155]. Among the most striking examples, TEs-derived ncDNAs have been involved in the rewiring of regulatory networks modulating flower development during rosid evolution, the C3 to C4 photosynthesis transition and seed endosperm development [155,240,241,242]. Endosperm formation involved RC/Helitron-derived CREs that rewired the genomic binding sites of MADS-box TF PHERES1 (PHE1) to ensure the proper expression of genes essential for seed development [242]. As the endosperm is the nourishing tissue feeding the embryo, this is reminiscent of the mammalian placenta formation, which suggests convergent evolution events.

### 5.3. TE-Derived RNAs Matter, but Is It Exaptation?

TEs are a major source of long non-coding (lnc)RNAs and small RNAs with *cis* and *trans* regulatory functions. TE-derived lncRNAs and small RNAs acting in *cis* have been well documented, especially in the RdDM or PTGS processes that were discussed above. TE-derived ncRNAs can also act in *trans* to regulate the expression of host genes at the transcriptional or post-transcriptional level. Besides, when embedded in a transcribed host mRNA, they can contribute to a plethora of mechanisms governing the mRNA outcome [158,243,244,245]. Although all these processes might not reflect genuine exaptation events, they clearly demonstrate that interaction between TEs and their host also occurs at the level of the RNA molecule (Figure 3B).

#### 5.3.1. SINE RNA

In mammals, the RNA Pol-III-transcribed *SINE* are well-studied TEs producing ncRNAs that have been involved in virtually all molecular processes regulating gene expression and organizing chromatin and nucleolus structure [20,246,247]. Although widely distributed in the human genome, *SINE* TEs, such as *Alu* elements (*Alus)*, are predominantly found in introns and 3′UTR of RNA Pol-II-transcribed genes [247]. Embedded in introns, *SINE* sequences can promote back-splicing events to form exonic circular RNAs [247]. In the 3′UTR of genes, *Alus* can form inverted-repeat (IR*Alu*) structures on mRNAs with regulatory functions. These 3′UTR IR*Alus* can influence host mRNA metabolism by controlling its subcellular localization, translation and stability through the recruitment of specific RNA-binding proteins [246,247]. Besides, 3′UTR *Alus* may be involved in alternative polyadenylation (APA) site usage to produce various mRNA isoforms [246], a process that is widely observed at the genomic level [248]. Remarkably, a similar process involving the *Drosophila pogo* DNA transposon was observed; pogo insertion in the 3′ UTR of the *CG11699* gene results in a shorter 3′UTR, elevated transcript levels and increased resistance to xenobiotic stress, suggesting an adaptive effect of the insertion [249].

#### 5.3.2. TE-Derived lncRNAs

There is compelling evidence, both in animals and plants, that TEs are a major source of lncRNAs, which are transcripts with a size greater than 200 bp and low protein-coding potential. In vertebrates, TEs account for up to 80% of lncRNA compositions. Comparative analyses performed in 40 plant species revealed more than 14,000 overlaps between TE and lncRNAs. While vertebrate lncRNAs appear to be lineage-specific, plant lncRNAs are generally poorly conserved between the same family species [158,244]. There is, however, a substantial number of TE-derived lncRNAs that are conserved among Brassicaceae species, suggesting conserved biological functions between these lncRNAs [250]. It is most likely that by nature, TE mobilization events have made the creation of lncRNAs a dynamic and fast-evolving process [251]. Thus, it seems fair to propose that TEs are an important source of lncRNA variability in genomes [252].

#### 5.3.3. TE-Derived Small RNAs

As mentioned above, TE-derived small RNAs have been widely described in plants, especially in the process of siRNA-mediated heterochromatinization (for recent reviews see also [158,244,245]). Different evolutionary scenarios depict how TE-derived small RNAs control host gene expression. For instance, transduplication or transduction processes might capture gene fragments, which will be potentially converted into small RNAs together with TE-associated sequences. Alternatively, upon TE transposition and subsequent immobilization, a TE or its fragment can become part of host gene. In both scenarios, small RNAs that are complementary to the host genes might be produced, which in turn will regulate its expression. We will focus here on a few examples of TE-derived small RNAs in complex plant genomes.

The interaction between TE-derived small RNAs and host genome is well documented in rice. First, the TE-related microRNA280 (miRNA820) downregulates in *trans* the host gene *OsDRM2*, which is involved in TE silencing, invoking the evolutionary arms race phenomenon between the TE and its host [253]. This is reminiscent of a previous report in *A. thaliana* describing *Athila* Ty3/Gypsy TE-derived siRNAs targeting *UBP1b*, which encodes a factor repressing *Athila* translation [254]. A second example described the two rice *WRKY45* allelic variants, *WRKY45-1* and *WRKY45-2*, which differ from each other by the presence or absence of two intronic *WANDERER*-type MITE TEs in opposite orientation, respectively [255]. The expression of *WRKY45-1* allele correlates with the accumulation of MITE-derived TE-siR815, which downregulates *SL1* expression by RdDM, conferring higher sensitivity to *Xanthomonas oryzae* (*Xoo*) infection. Conversely, *WRKY45-2* expression confers improved resistance. The negative effect of TE-siR815 was confirmed by creating transgenic plants overexpressing *WRKY45-1* with deleted *TE-siR815* sequence that showed enhanced resistance, similar to *WRKY45-2*-overexpressing plants [255]. Third, the stowaway-like *MITE* (*sMITE*) TE is integrated in the 3’-UTR of the *Ghd2* gene, which promotes *Ghd2* translational repression in a siRNA-dependent manner interfering with panicle development and grain yield [256]. *Ghd2* is important for agronomical purposes, as it controls grain number, plant height and heading date. Comparative analyses revealed that *sMITE* is present in *O. rufipogon*, a wild relative of cultivated Asian rice [256], which suggests a positive selection event. Moreover, Shen and colleagues showed that several rice genes display MITEs in their 3′UTR, implying that *MITE*-driven translation repression could be a widespread mechanism [256].

In maize, captures of gene fragments by TEs can lead to evolutionary conflicts [215]. SiRNA crosstalk can occur between donor genes and TEs carrying the gene fragments, making donor genes more prone to accumulating siRNAs, to be DNA methylated and transcriptionally repressed. While this would be particularly true for translocated donor genes that are under low selective pressure, it is not the case for donor genes with important function that maintain high expression level with lower DNA methylation/siRNA levels, suggesting that capture of gene fragments could provide selective advantage to TEs [215]. Finally, in bread wheat (*Triticum aestivum* L.), a recent study showed that class II MITE and Mariner TEs are important sources of miRNAs with a potential role in plant immunity [257]. Particularly, the authors identified Tae_miR1436-1, which downregulates the accumulation of the Metallothionein-3-like (TaeMt3) protein to induce cell death as an efficient immune response against the powdery mildew fungus *Blumeria graminis f.sp tritici*. *Tae_miR1436-1* is conserved in the *triticum* lineage, but not in other plant species [257]. Whether this case is a genuine event of exaptation or not is still a matter of debate, but it undoubtedly demonstrates the major evolutionary force of TEs to rewire host gene expression at the RNA level.

#### 5.3.4. Biological Function of TE-Derived ncRNAs

Briefly, in animals, TE-derived lncRNAs have been related to several developmental processes. The maintenance of pluripotent embryonic stem cell program involves ERV-lncRNAs and LINE RNAs in humans. The mouse and human SINEUP lncRNAs have been involved in brain development by positively regulating the translation of factors essential for brain development. Finally, the LINE- and SINE-derived *Xist* is most likely the most-studied lncRNA, with a major role in the process of X-chromosome inactivation in female eutherian mammals [158,243]. In plants, the transcription of several TE-derived lncRNAs and small RNAs is induced by environmental stresses or phytohormone treatments. TE-derived small RNAs play a role in abiotic and biotic stress responses, plant development, hybridization barrier and of course, TE silencing [244,245].

Considering that overall, plant lncRNAs are involved in many biological processes, such as chromatin topology, transcriptional regulation, alternative splicing and small RNA buffering, all together regulating fundamental processes of plant development such as flowering time or stress perception [244,245], it is most likely that plant-TE-derived lncRNAs greatly contribute to these mechanisms as well.

## 6. How to Identify TE-Derived Sequences?

A stepwise approach must be implemented to define true exapted TE events. Some steps, such as identification, functional validation and conservation among close relative species are requisite and common to both types of exapted sequences (coding and non-coding ETE). Others, for instance, expression analyses when referring to ETE genes or exaptation of TE-derived RNAs, are more specific to one type of event [155,258].

### 6.1. Identification of TE-Derived Sequences

Constant improvements in sequencing technologies together with drastic reduction of sequencing costs have allowed large-scale genomic projects aimed at determining the genome of hundreds of species. It is nevertheless important to mention that careful genomic annotations, with a special attention to DNA repeats, are a prerequisite for the identification of TE-derived sequences. Indeed, identifying an exapted TE among a pool of “regular” TEs can be seen as looking for a needle in a haystack. Thus, meticulous computational analyses must be undertaken.

For the identification of ETE genes, one can integrate a suite of common properties including loss of mobility—absence of flanking terminal repeats such as TIRs or LTRs, sequence similarity with functional proteins (like transposases), low copy number, a conserved genomic location among sister/cousin species (synteny), evidence of active transcription and purifying selection of key codons [25]. Using similar approaches, several putative ETE genes were identified in *A. thaliana* [259]. A particularly challenging aspect is to establish the evolutionary history of an ETE gene family in order to determine the number and timing of exaptation events, and the identity of the ancestral genomes in which they arose. Such an approach was designed to resolve the phylogenetic context and timing of exaptation events and subsequent patterns of *MUSTANG* and *FAR1/FHY3* diversification [170].

To identify ETE non-coding elements acting as CREs, it is important to first annotate TE-derived sequences in the genome. A recent study described a new approach to find old and degenerated TEs in *A. thaliana*. Based on a *k-mer* strategy, the authors suggested that half of the genome would originate from TEs, which is significantly more than previously anticipated [260]. Then, the identification of TE-derived sequences localized near genes, for instance in their promoter, needs to be realized. Bioinformatic tools can be used to predict the presence of binding motifs in these sequences [261]. Baud and colleagues found that among the degenerated TE sequences, a significant number corresponded to TFBS [260]. Wet lab approaches based on chromatin immunoprecipitation followed by sequencing (ChIP-seq) can be achieved to identify TFBS in TE-derived sequences at the genomic scale [235,262,263]. Importantly, some TE-derived CREs can also act as distal regulators such as enhancers (Figure 3B). Moreover, ETE non-coding DNA elements can be involved in other chromatin-related processes, such as, for instance, genome organization and compartmentalization.

### 6.2. Functional Validation

It is subsequently important to perform functional experiments when evaluating gene regulatory roles of TEs. For this purpose, in vivo enhancer assays, such as luciferase-based reporter experiments, can validate candidates [193]. However, these experiments are limited by the fact that they dissociate the exapted TE from its native chromosomal context, which can render the establishment of a direct causal link between the *cis*-regulatory activity of the TE and the endogenous gene expression difficult. Another approach to validate the role of a specific non-coding ETE sequence on host gene regulation is to carry out a loss of function experiment. Recent development of precise genome editing technologies such as CRISPR-Cas9 system allows one to answer this question [264,265,266].

#### 6.2.1. Conservation among Closely Related Species

Microsynteny analyses are an essential step to define true ETE events. They rely on analyzing the genomic environment of TE-derived sequences among sister and more distant cousin species of a specific lineage to highlight evolutionary conservation. However, these analyses can be tricky when dealing with old events. Indeed, synteny can be interrupted by genomic rearrangements, making it difficult to determine the conservation of exapted events among distantly related species. This demonstrates the difficulty in drawing a conclusion based on a single feature. Furthermore, evidence of purifying selection events for conserved key codons within orthologous ETE genes is another essential feature reinforcing the confidence in exaptation events. Nevertheless, it is likely that many ETE sequences have escaped detection due to lack of solid evidence of conservation among closely related species.

#### 6.2.2. Expression of ETE Genes or TE-Derived RNAs

Although not sufficient by itself, evidence of active transcription is a good clue of TE exaptation if combined with other features described above. Nevertheless, in some cases, it might be difficult to detect signs of expression. Indeed, it is important to bear in mind that the ETE sequences could be expressed at a low level or display spatiotemporal expression patterns, as they are often cell-lineage- or tissue-specific [267]. The new method CELLO-seq, which is based on long-read RNA sequencing, allows the detection of TE expression at single cell resolution, considering allelic and isoform variants [268]. In addition, the detection of TE-host fusion mRNAs must rely on robust detection methods integrating alternative splicing events. An excellent review synthetizes the different technologies and computational approaches that can be undertaken to detect TE expression [269].

#### 6.2.3. Combining Computational and Wet Lab Approaches

The best way to highlight the impact of ETE events on host genome is to combine different approaches, such as computational genomics, transcriptomics (RNA-seq), genome editing and multiple chromatin-related analyses allowing the genome-wide mapping of TE-derived CREs or TF-like ETE proteins (ChIP-seq, DAP-seq, ATAC-seq, CAGE-seq…). Besides, cell biology and biochemical studies are essential complementary approaches to fully picture the functional role of ETE sequences. Although they might not be genuine ETEs, it is most likely that TEs and other DNA repeats overall contribute to 3D genome organization. Chromosome conformation capture (3C) analysis, such as Hi-C, studies subnuclear chromatin organization and compartmentalization, which can give new insights into the role of TEs in these processes. Mammalian class I TEs contribute to the species-specific subgenomic binding of the insulator protein CCCTC-binding factor (CTCF), which differentially impacts DNA looping and gene expression at the species level [270,271]. Likewise, 3C-based 4Tran experiments in human and mice captured long-range interactions between ERVs and host genomes, with a potential role in regulation of gene expression [272].

#### 6.2.4. Long-Read-Based New Sequencing Technologies

As already mentioned, the fact that ETE sequences derive from highly repeated TEs can make their identification strenuous. For instance, upon a recent exaptation event, it might be difficult to differentiate the functional ETE gene from homologous copies that did not enter positive selection. Long read sequencing technologies, such as Pacific Biosciences (PacBio) or Oxford Nanopore sequencing, can identify whole TE or TE-derived sequences in complex genomes, as described recently in a genomically instable *A thaliana* line depicting chimeric TE/gene fusion event [273]. Such technologies can also be used to identify ETE genes. Besides, by performing long read nanopore RNA sequencing, it is now possible to capture full-length TE transcripts [274], giving important information about TE—and potentially ETE—expression in specific genetic background or environmental conditions.

## 7. Conclusions

In the future, the advent of large-scale genome sequencing combined with long read sequencing technologies will undoubtedly contribute to the identification of new TE-derived sequences with potential selective advantage for the host. Besides, pursuing our understanding of molecular processes involved in the regulation of epigenetic pathways and TE silencing will also be crucial. Altogether, these studies will undoubtedly contribute to unravelling the complex interplay between TEs and their host.

## Data Availability

Not applicable.
